# A Time Sequence Images Matching Method Based on the Siamese Network

**DOI:** 10.3390/s21175900

**Published:** 2021-09-02

**Authors:** Bo Tao, Licheng Huang, Haoyi Zhao, Gongfa Li, Xiliang Tong

**Affiliations:** 1 Key Laboratory of Metallurgical Equipment and Control Technology, Ministry of Education, Wuhan University of Science and Technology, Wuhan 430081, China; taoboq@wust.edu.cn (B.T.); ligongfa@wust.edu.cn (G.L.); 2 Hubei Key Laboratory of Mechanical Transmission and Manufacturing Engineering, Wuhan University of Science and Technology, Wuhan 430081, China; liuying3025@wust.edu.cn (H.Z.); tongxiliang@wust.edu.cn (X.T.)

**Keywords:** similarity, image pair, the Siamese network, correlation matrix, comparison

## Abstract

The similar analysis of time sequence images to achieve image matching is a foundation of tasks in dynamic environments, such as multi-object tracking and dynamic gesture recognition. Therefore, we propose a matching method of time sequence images based on the Siamese network. Inspired by comparative learning, two different comparative parts are designed and embedded in the network. The first part makes a comparison between the input image pairs to generate the correlation matrix. The second part compares the correlation matrix, which is the output of the first comparison part, with a template, in order to calculate the similarity. The improved loss function is used to constrain the image matching and similarity calculation. After experimental verification, we found that it not only performs better, but also has some ability to estimate the camera pose.

## 1. Introduction

Judging the relationship of an image pair is a common issue in the field of computer vision. It has an important effect in SfM (structure from motion), image retrieval, pose estimation, and stereo match. Image matching [1,2,3] refers to judging the relationship between image pairs by identifying the same or similar scenes, objects, shapes, and semantics of two images. Image matching is a basic vision technology, and the result of image matching directly influences the effect of SLAM (simultaneous localization and mapping), 3D reconstruction, and scene understanding. Statistically, above 40% of vision perception tasks rely on precise and effective image matching. These applications include computer vision, pattern recognition, military security, medical diagnosis, and so on.

With the rapid development of image matching technology, many approaches based on the Siamese network [4,5] are proposed. Due to its unique architecture, the Siamese network performs much better compared to other networks in face recognition [6,7], instance segmentation [8], object tracking [9,10,11,12], and so on. It is considered as a promising network in image matching. In this paper, the raw data are time sequence images. We make random samples in sequence images to obtain an image pair. A label is constructed by the time interval of images in an image pair. The value range of the label is between 0 and 1. Details of the label construction are presented in Section 4. A label indicates the similarity of images in a pair. We can judge whether the image pair is matched by similarity. The method can easily and conveniently construct a Siamese network dataset.

In conclusion, our major contributions are as follows:

1. Propose a Siamese network for image matching;
2. Propose a convenient and rapid approach to making the dataset;
3. Verify the effect of the Siamese network in image matching.

This paper is organized as follows. Section 1 introduces the application, background, and significance of image matching. Section 2 introduces the related work about the Siamese network and image matching. Section 3 describes our proposed method and architecture in detail. Section 4 shows the details of the experiment. In Section 5, conclusions are presented and future work is discussed.

## 2. Related Works

Image matching is a classical issue in the image process. In the past 10 years, CNNs (convolutional neural networks) have achieved great progress in image classification, object detection, semantic segmentation, and other fields with a single image. However, most CNNs find it difficult to establish data association with multiple images. Classical CNN architecture is unsuitable for image matching. At present, the major obstacles in image matching are the effects of environmental changes. These changes are caused by factors such as the viewpoint, scale, and illumination. Some traditional algorithms were proposed to deal with these obstacles. There are also some typical algorithms, such as SIFT [13], SURF [14], ORB [15], and the color histogram algorithm [16]. These traditional algorithms use different features to achieve image matching. Based on traditional ones, some improved algorithms were also proposed, such as CSIFT [17], BRISK [18], ORB [19], FREAK [20], LBP [21], and stereo key-point matching [22]. The improved algorithms mentioned are simplified in feature representation in order to improve the overall computational efficiency. However, the traditional algorithms depend on the design and extraction of handcraft features, which are time-consuming, susceptible to environmental interference, less robust, and lacking generalization in practice.

The Siamese network is different from classical CNN architecture. It takes dual inputs and has two feature extractions that share weights. The Siamese network can establish the data association between dual inputs by some fusion operations in the output of feature extractions, such as element subtraction, weighted summation, and so on. The Siamese network was first proposed by LeCun et al. [23]. LeCun applied the Siamese network to handwriting signature verification and achieved the best performance in that time. Then, Hinton et al. first combined it and deep learning to face recognition. With the popularity of deep learning, more and more researchers have combined deep learning and the Siamese network. Luca et al. [24] replaced full-connection layers with convolutional layers to propose FC-Siamese (fully convolutional Siamese) networks. Forian et al. proposed FaceNet [25]. Held et al. [26]. explored the effect of the Siamese network in few-shot learning. Some researchers have made some significant works for the Siamese network in representation learning [27,28], and the Siamese network also became an important direction in self-supervised learning [29,30,31].

## 3. Method

**Figure 1:**
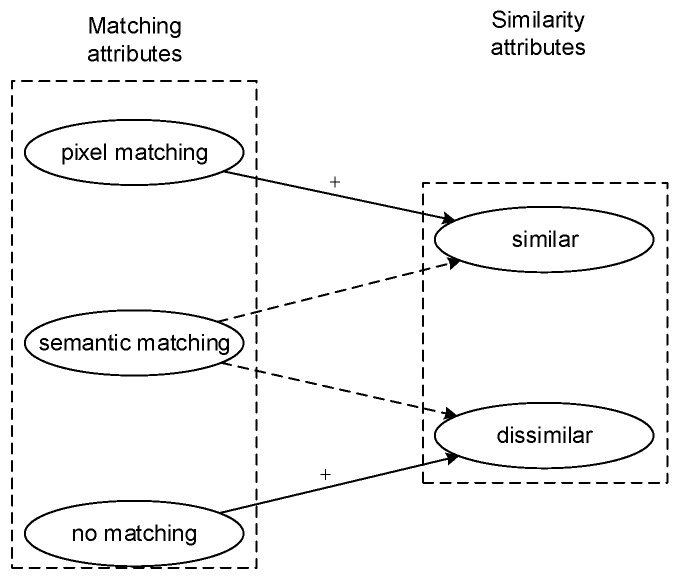
Relationship between match attribution and similarity attribution.

In image matching, the input image pair has two attributes: similarity and matching. The matching attribute has 3 states to select: pixel matching, semantic matching, or mismatching. The similarity has 2 states to select: similarity or dissimilarity. We defined images in inputs as the original image and the matching images, respectively. Pixel matching requires that pixels in the original image can be found in the matched image with the same intensity and geometric relationship. Semantic matching refers to the establishment of a correspondence domain between two images based on semantic consistency [32]. In other words, semantic matching relies on the semantic information of the pixels rather than the physical information (intensity and geometric relationships). The physical information of a pixel is susceptible to environmental changes, but the semantic information is relatively stable. Therefore, pixel matching is only suitable for image matching in static environments, whereas semantic matching is suitable for image matching in both dynamic and static environments. Mismatching means that pixels in the original image and the matching image are different in both physical and semantic domain. Similarity is our final aim. In Figure 1, pixel matching and mismatching can directly judge between similar or dissimilar, which is indicated by solid lines. By contrast, semantic matching finds it hard to discriminate between similar or dissimilar, which is indicated by dotted lines.

In fact, pixel matching is a special case in semantic matching. Due to the fact that motion brings translation and rotation to the object, the match image is wrapped in pixels. When matching image is wrapped with camera motion, pixel matching degenerates to semantic matching. Semantic matching has semantic invariance, which is robust. However, the relationship between semantic matching and similarity attribute is not formulated. The reasons are as follows:

- Semantic information is hard to measure;
- Boundary between similar and dissimilar pairs is hard to define.

The most widely used criterion is Euclidean distance. Compared to the Euclidean distance, the probability distribution greatly reduces the influence of local features for image matching. The probability reflects the motion trend in physical and consistency in mathematics. Thus, it retains local features and global features in high dimension space. For the boundary, we designed a neural network to search in a high dimension and output a value to describe similarity attribute.

In this paper, we built 16 anchors uniformly in each image of input pairs. Full convolutional network was used as a feature extraction. Feature extraction was applied to inputs to obtain feature maps, which are composed of 16 anchors. Each anchor in the feature map represents a local feature in the inputs. We called it a patch. Due to the complexity of the distribution, 16 anchors are few. It cannot construct a precious explicit probability distribution.

We turned to construct a 16 × 16 correlation matrix. It describes the correlation between each patch in the original image and all patches in the matching image. Then non-linear transformation operated on correlation matrix to obtain correlation distribution *φ*. When the original image and matching image are the same image, the correlation matrix represents a unique form: identity matrix, which remains the same formulation in correlation distribution. We defined the correlation distribution with the attribute as label distribution *ζ*. In general cases, the original image and the matching image had some differences, which are reflected in *φ* and lead to *φ* not being equal to *ζ*. Therefore, differences, which are negatively related to the similarity, between *ζ* and *φ*, are good criterion.

### 3.1. The Siamese Network

The Siamese network’s attributes indicate that it can eliminate random noise and find correlation. For each input, the Siamese network has an instance of feature extraction, and instances share the same weight. In parallel architecture networks, multiple inputs are fed to the parallel blocks. Parameters in parallel blocks are different. Thus, parallel architecture reserves the attribute of random and enforces inputs correlation. Random is harmful, which introduces noise to the system. In series architecture network, single input is required and only one data flow is supported. During training, all inputs are considered independent and identically distributed. Thus, series architecture is stable without random noise. Single input means that it is hard to find the correlation in dataset.

As Table 1 shows, the Siamese network combines pros of parallel and series architecture using hybrid architecture. The front part constructed a parallel network with two feature extraction instances that are shared weights. The latter part of it is a series network to evaluate the similarity.

The Siamese network can avoid mutual interference of inputs and maintain the structural similarity of the input data. In the AI (artificial intelligence) fields, the Siamese network belongs to comparative learning or metric learning. The base principle for comparative learning is boundary measurement. It needs one input as a template, which contains much prior knowledge. Another input is compared to the template. The network can find right boundaries to distinguish different attributes by comparison. Based on that, the network learned the representation.

For that purpose, the dataset is a key. Fitting dataset makes network learned latent representation and improper dataset leads to bad parameters. In the dataset, positive samples were the image pairs with a similarity, and negative samples were the image pairs with enough difference. Negative samples are more than three times as large as positive samples in order to keep data distribution in a suitable domain.

Correlation matrix and correlation distribution *φ* needed to meet the following conditions:

1. When the input images are the same, *φ* is equal to *ζ*;
2. The stronger the correlation of the corresponding patch, the greater the probability value;
3. In probability, the value range of each element in *φ* is between 0 and 1 (including 0 and 1), and the elements in each row accumulate to 1;
4. The more similar the input image pairs are, the more similar *φ* is to *ζ*.

As for condition 1, the output of the feature extractor in the Siamese network was subtracted to generate a correlation matrix. Rows and columns in matrix represent the position coordinates of the patch in the original image and the matching image, respectively. The element’s value in matrix represents the correlation. The stronger the correlation, the lower the value. When the original image and matching image are equal, the correlation is the strongest, and the value is 0. In order to satisfy condition 2, the Gaussian function Formula (1) was used to transform the output space and enhance nonlinearity. In order to satisfy condition 3, the softmax function was used, as in Formula (2).

**Figure 2:**
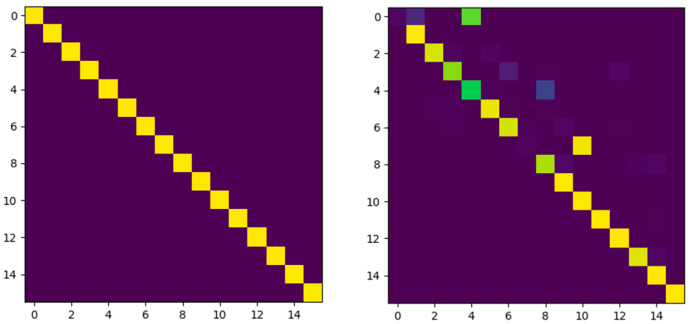
The label distribution *ζ* and the correlation distribution *φ*. The intensity of the color indicates the strength of the correlation; the strongest is 1, and the weakest is 0. The ordinate indicates the position of each patch in the original image, the abscissa indicates the position of each patch in the matching image, and the row indicates the correlation between a certain patch in the original image and all patches in the matching image. If the original image and matching image are equal, each row is unimodal. Rows being multimodal means that the patch is ambiguous and hard to match.

As shown in Figure 2, alongthe column direction of the correlation matrix, each row in the correlation matrix was normalized. Since normalization can transform rows into probability distributions, the correlation matrix becomes a correlation distribution *φ*, which is composed of a series of probability distributions. Each row in *φ* represents a classification result. The input of this classification is a patch of the original image, and categories are all patches in the matching image.

The variable *x* in the Gaussian function represents each element in the correlation matrix, which is linear. The parameter *σ* in the Gaussian function is related to the scale of the input and affects distribution concentration. A lower *σ* can strengthen the positive correlation and make unimodality. A higher *σ* can strengthen the negative correlation and make multimodality. The express $exp(x_{i})$ in Formula (2) is equal to Formula (1); thus $x_{i}$ is the same as $-{\left(\frac{x}{2\sigma }\right)}^{2}$. The parameter k is the number of columns. As for Figure 2, k is 16.

(1) $$\mathrm{Gassian}(x)=exp(-{\left(\frac{x}{2\sigma }\right)}^{2})$$

(2) $$\mathrm{Gassian}(x)=exp(x_{i})$$

*φ* can reflect the local correlation between the original image and the matching image. It cannot solve the global correlation between the original image and the matching image directly. The relationship between similarity and *φ* is more complicated. *S* and *φ* are negatively correlated in value, and the structure relationship between *S* and *φ* is closely related. We decided to use conditional probability to describe the relationship, which is non-linear, as in the Formula (3) shown.

(3) P(S|φ,ζ)=Similar_network(D(φ,ζ))

The Similar network is a neural network for solving the conditional probability P(S|φ,ζ). The Similar network is designed as a simple fully connected network. D(φ,ζ) denotes the result of the improve KL (Kullback–Leibler) divergence, which is discussed in next section. The activation function uses the tanh function with bias of 0. The tanh function is shown in Formula (4). The variable *x* represents the output of fully connected network. We took the absolute value of the tanh function as the similarity to ensure that the value range of the similarity is same to the value range of the label.

(4) $$\tanh(x)=\frac{exp(x)-exp(-x)}{exp(x)+exp(-x)}$$

**Figure 3:**
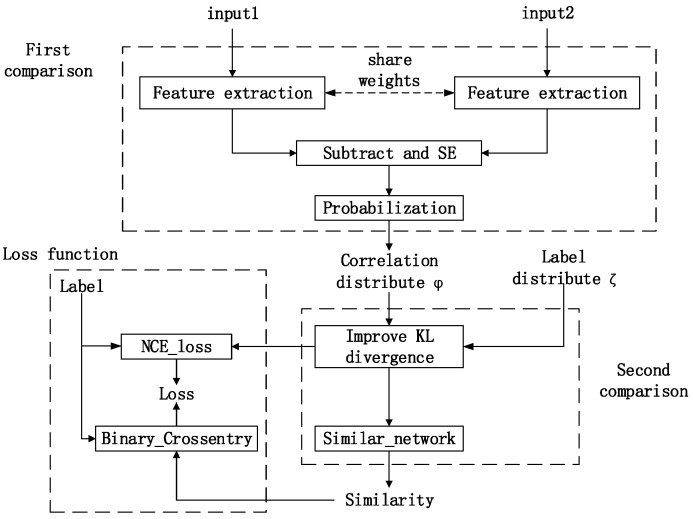
Network architecture.

The overall architecture of the network is shown in Figure 3. In our architecture, there were two different comparison parts. The first part was to compare the original image with matching images. The second part was to compare correlation distribution *φ* with label distribution *ζ*. The first part aimed to find correlation of inputs and generate the correlation distribution *φ*. The second part aimed to calculate the similarity by correlation.

### 3.2. Improved KL Divergence

In Siamese network, the most classic contrastive loss function is Info NCE (Info Noise-Contrastive Estimation) loss function [33]. For similar or dissimilar inputs, the Info NCE loss function can guide the network to reduce or increase differences, respectively. Therefore, the key of the Info NCE loss function lies in the semantic information difference measurement. Some classic measurement functions, such as Euclidean distance and cosine distance, are naive. There is a lack of comparison of the overall difference in the input, and it is susceptible to the influence of some special features, which leads to larger deviations. This paper proposes an improved KL divergence as the similarity measurement function to compare the similarity of the inputs.

For probability distribution, KL divergence is typically used to measure the differences between the two probability distributions, as in Formula (5). However, KL divergence is sensitive to the distribution p(*x*), but insensitive to the distribution q(*x*). In distribution p(*x*), the part near the peak region can be exactly estimated., and the probability of the part that is far from the peak region approaches 0. The position on the distribution p(*x*) represents the space coordinates of the corresponding patch. According to object motion, the closer the space positions are to patch, the stronger the similarity is. Ignoring the spatial coordinates increases the error.

(5) $$D_{KL}(p||q)=\int p(x)\ln\frac{p(x)}{q(x)}dx$$

Due to being unimodal, the influence of the spatial coordinates is concentrated near the peak, and the value in non-peak area is zero. Therefore, we use coordinates to weigh the difference value, and only the value near the single peak of the distribution *φ* and *ζ* are affected, whereas the other positions are masked. Similarity between the distributions is measured by integration. Since the coordinates on the image have different directivities, we weigh *φ* according to different directions. The Manhattan distance is used to calculate the total distance, as shown in Formula (6).

(6) $$D(\varphi ,\zeta )=\int x\ln\frac{\varphi (x,y)}{\zeta (x,y)}dx+\int y\ln\frac{\varphi (x,y)}{\zeta (x,y)}dy$$

Parameters *x*, *y* represent the coordinate *φ*(*x*, *y*), and *ζ*(*x*, *y*) represents the probability that the *x*-th part in the original image matches the *y*-th part in the matching image, based on the distribution *φ* and the distribution *ζ*, respectively. The lower the D scores, the closer *ζ* is to *φ*, and the higher the D scores, the larger the difference between *ζ* and *φ*.

### 3.3. The Channel Attention

Channel attention mechanism refers to applying attention mechanism on channel dimension. The channel in feature map represents various semantic information. While each channel is independent, it increases difficulty to feature fusion. Channel attention can promote the quality of feature fusion, producing more advanced features, which are important factors in the image matching process. It learns channel weight distribution, which contains channel correlation. Each channel multiply corresponding weights in order to make feature fusion.

**Figure 4:**
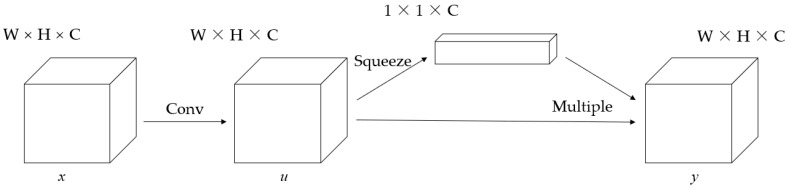
SE unit. W represents the vector width. H represents the vector height. C represents the number of vector channel. The input *x* is convolved to obtain the feature *u*. The weight is generated by compressing u into the channel dimension through the squeeze process. Finally, the weighted feature y is obtained by multiplying the weight by the corresponding channel.

In this paper, we adopted SE (squeeze and excitation) architecture to achieve channel attention mechanism [34]. The SE unit could focus on important features and ignore unimportant features. A SE unit is shown in Figure 4. It has a series of convolution layers and a squeeze layer. The squeeze layer, which includes global average pooling and global maximization pooling, generates weight.

### 3.4. The Design of Loss Function

In general, loss function in the Siamese network is characterized by high cohesion and low coupling. Loss function can maximize inter-class differences and minimize intra-class differences. In this paper, we designed a loss function, which is shown in Formula (7)

(7) loss=BCE(l,P(S|φ,ζ))+L

Our designed loss function consisted of two parts. The first part was the classic BCE (binary cross-entropy) function, which is widely used in binary classification tasks. The BCE function requires *l* and P(S|φ,ζ) as inputs, where *l* is a label that decides whether the original image matches the matching image (*l* = 1) or not *(l* = 0), and P(S|φ,ζ) is defined in Formula (3). Since P(S|φ,ζ) is the result of Similar network, the BCE function is mainly trained on the parameters of Similar network. The second part is a contrastive loss function, which is shown in Formula (8):

(8) L=(1−l)D+l{max(margin−D,0)}

where D is a function defined in Formula (6), the parameter *l* is same as the *l* in BCE function, and *margin* represents the boundary of differences. Only when the difference value is within a certain range can the loss be optimized. If the difference is out of range, a constant is set and no optimization occurs. In the experiment, the margin was set to 1. The L required label and correlation distribution *φ* for learning. Since *φ* are results of feature extraction, *φ* focused on feature representation, and L mainly trained the parameters in the feature extractor.

## 4. Experiment

In experiment, the dataset was some color image videos from KITTI [35], and the input image was resized to 64 pixels on the horizon and vertical. Positive and negative samples were constructed by chronological order. The image pairs with time interval less than n were taken as positive samples and labeled as 0. The ones greater than n were taken as negative samples and labeled as 1. The selection of hyper parameter n was related to the correlation of image pair: If n too small, not enough correlation would be learned; otherwise, unnecessary errors will be introduced if n is too large. Fewer positive samples could not learn correlation precisely. Fewer negative samples introduced unnecessary errors. According to the instructions given by KITTI dataset, one interval was 0.1 s on average. We decided to set n to 5. When n was greater than 5, the change caused by camera motion was apparent and the correlation of image pairs was low.

By means of sampling, a dataset was constructed. The dataset contained positive and negative sample pairs. The positive sample pairs were guaranteed to account for approximately 25% of the dataset, and the negative sample pairs were far greater than the positive sample pairs. In the Siamese network, feature extraction adopted the full convolutional network architecture and the similar network adopted 1 layer of full connection network. The network architecture is shown in Table 2. Due to the fact that the padding in the convolution process can affect the scale invariance, all the convolution layers had no padding.

In the experiment, the model was trained on NVIDIA GTX 1080 Ti GPU with 12 GB memory. The inference time for singe image was 10 ms, and the total parameter size was 20 M. The accuracy was 89.2%. We compared a variety of networks with different architectures, as shown in Table 3.

We compared the effects of improving the KL divergence and SE unit. As for the SE unit, it was found that, after using SE structure in the network training process, the value of loss function decreased more stably and the convergence speed was faster. With the improved KL divergence, the loss value could be maintained at a lower level and had better generalization performance. In conclusion, SE unit and improved KL divergence can improve the matching accuracy and processing speed in the Siamese network.

We compared the performance of our method, triple Siamese network, and sHybirdNet, in different scene datasets. These scenes included street, urban, and highway. Different scenes have different characteristics. The results are shown in Table 4.

- Highways have fewer features and different features are highly similar (e.g., lanes);
- Cities have more features and some features have random motion (e.g., pedestrians);
- Streets have more distinguishable features and most of the features have small position changes in the image (e.g., buildings).

**Table 4:** Comparison of different approach.

Dataset Scene	Siamese + SE Unit + Improved KL Divergence (Our Method)	Triple Siamese Network [36]	sHybridNet [37]
Streets	89.2	82.5	75.2
Cities	78.2	74.6	65.4
Highways	69.9	68.2	54.2

Through experiments, we found that all networks work better in street scenes and worse in highway scenes. Our proposed method performs much better than sHybirdNet in all scenes, with an improvement greater than 10%. Compared to triple network, our proposed method also has some advantages.

**Figure 5:**
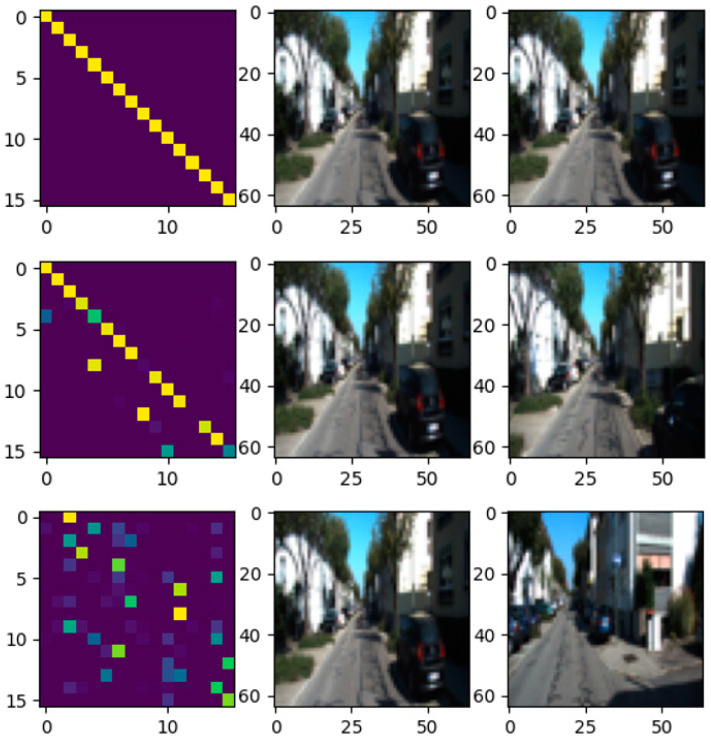
Correlation distribution *φ* and input image: top, *φ* with identical input images, and the output from the similar network is 1 × 10^−6^; middle, *φ* with similar input images (difference between 5 frames), and the similar network output is 0.653; bottom, *φ* with completely different input images (difference between 50 frames), and the similarity is 0.998.

As shown in Figure 5, the correlation of the input image pairs is manifested in the structure and intensity. The intensity indicates the similarity of each patch in image pairs. In the higher similarity of image pairs, there are more bright patches in *φ*, which indicates that most patches in the original image can find the corresponding matching patches in the matching images. The more similar the image pairs are, the more patches with high correlation in *φ*. The structure of the *φ* represents the position relationship of each block in the original image and the matching image. The higher the similarity of image pairs, the closer *φ* and *ζ* are in structure.

**Figure 6:**
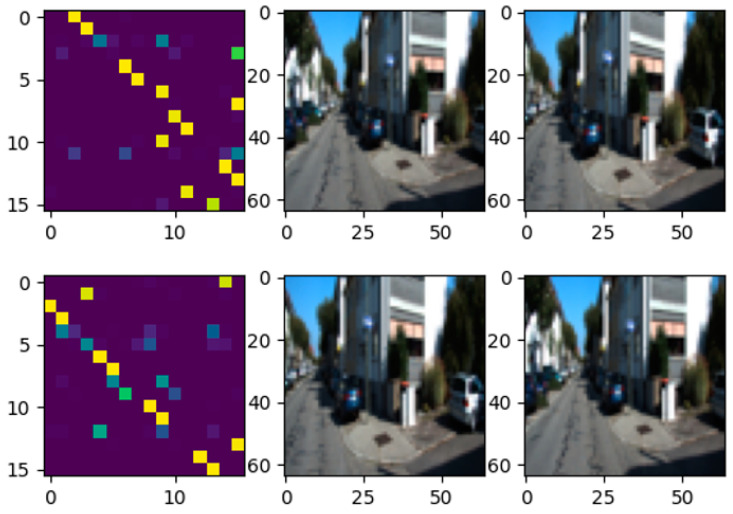
Correlation distribution *φ* in the case of camera rotation: top, the change in the *φ* when the camera is rotated to the right; bottom, the change in the *φ* when the camera rotates to the left.

We can obtain more information from the structure of *φ*, such as in Figure 6. When the camera rotates to the right or left, the corresponding distribution can shift to the right or left without breaking the structure as much as possible. The structure and intensity of *φ* can be analyzed by the similar network, which is sensitive to changes in *φ*. Therefore, the motion changes between the original image and the matching image can be further inferred through the similar network.

## 5. Conclusions

In this paper, an image matching method based on the Siamese network is proposed. We use probability distribution to model the input. The network can distinguish the similarity and difference in input through a comparison. Based on the method, the network outputs the similarity between images and determines the matching relationship between images. Based on the basic Siamese network, an improved KL divergence is used to evaluate the distribution difference, which comprehensively considers the influence of the position. It also introduces the SE unit in order to accelerate the convergence and keep it stable in the training process. It uses convolution to divide the image into multiple image patches for matching, which is different from directly matching the whole image. By comparing label distribution *ζ*, it keeps the consistency and global constraint on the matching results. In addition, the correlation distribution *φ* can estimate the basic motion of the camera.

In this paper, the input is resized to 64 × 64. With a larger input scale and more complex feature extractor, fine features can be extracted; it also generates more patches, which increases the difficulty of matching. The similar network is constructed by a simple fully connected layer. More complex network structures can measure the similarity in different metrics. The correlation matrix is obtained by subtraction. In addition, a more accurate correlation matrix can be obtained by more advanced methods, such as the graph network method.

In future work, we will use a larger scale input, combined with the principle of target detection, to screen blocks in order to obtain interested blocks, so as to greatly reduce the complexity caused by the increase in input scale.

## Figures and Tables

**Table 1:** Pros and cons of Siamese network.

	Parallel Network	Series Network	Siamese Network
Pros	Keep correlation	Stability	Eliminate random noise Keep correlation
Cons	Introduce random noise	Ignore correlation	Correlation hard to model

**Table 2:** Network architecture.

Feature Extraction	Similar Network
Input 64 × 64 × 3	Input 16 × 16
7 × 7 conv 16 BN ReLU valid	Flatten
5 × 5 conv 32 BN ReLU valid	1 Dense Tanh
3 × 3 conv 256 BN ReLU valid	
3 × 3 conv 128 BN ReLU valid	
3 × 3 conv 128 BN ReLU valid	
2 × 2 maxpool	
3 × 3 conv 256 BN ReLU valid	
3 × 3 conv 512 BN ReLU valid	
3 × 3 conv 256 BN ReLU valid	
3 × 3 conv 256 BN ReLU valid	
3 × 3 conv 512 BN ReLU valid	
3 × 3 conv 256 BN ReLU valid	
3 × 3 conv 256 BN ReLU valid	

**Table 3:** Result of different networks.

Name	Accuracy (%)
Siamese	84.2
Siamese + improved KL divergence	88.6
Siamese + SE unit + improved KL divergence	89.2

## Data Availability

Data sharing is not applicable to this article.
